# Case for diagnosis. Hyperpigmented and excoriated papules and nodules in a diabetic patient^⋆^^⋆⋆^

**DOI:** 10.1016/j.abd.2020.03.015

**Published:** 2020-09-13

**Authors:** Catalina Hasbún, Mauricio Sandoval, Sergio González-Bombardiere

**Affiliations:** a School of Medicine, Faculty of Medicine, Pontificia Universidad Católica de Chile, Santiago, Chile; b Department of Dermatology, Faculty of Medicine, Pontificia Universidad Católica de Chile, Santiago, Chile; c Department of Pathology, Faculty of Medicine, Pontificia Universidad Católica de Chile, Santiago, Chile

**Keywords:** Collagen diseases, Diabetes mellitus, Kidney failure, chronic, Paraneoplastic endocrine syndromes

## Abstract

Reactive perforating collagenosis is a rare perforating dermatosis clinically characterized by intensely pruritic hyperpigmented papules, plaques, and nodules with a central keratotic plug. Histopathology reveals transepidermal elimination of collagen fibers. Its pathophysiology is still under investigation, but the acquired form has been linked to systemic conditions such as diabetes mellitus and chronic kidney disease. However, it has also been described as a paraneoplastic syndrome. The authors present the case of a 65-year-old diabetic patient in which a myeloproliferative neoplasm was suspected.

## Case report

A 65-year-old diabetic female with poor metabolic control (HbA1c 14.9%) presented with a two-month history of pruriginous lesions on the trunk and extremities.

**Figure 1:**
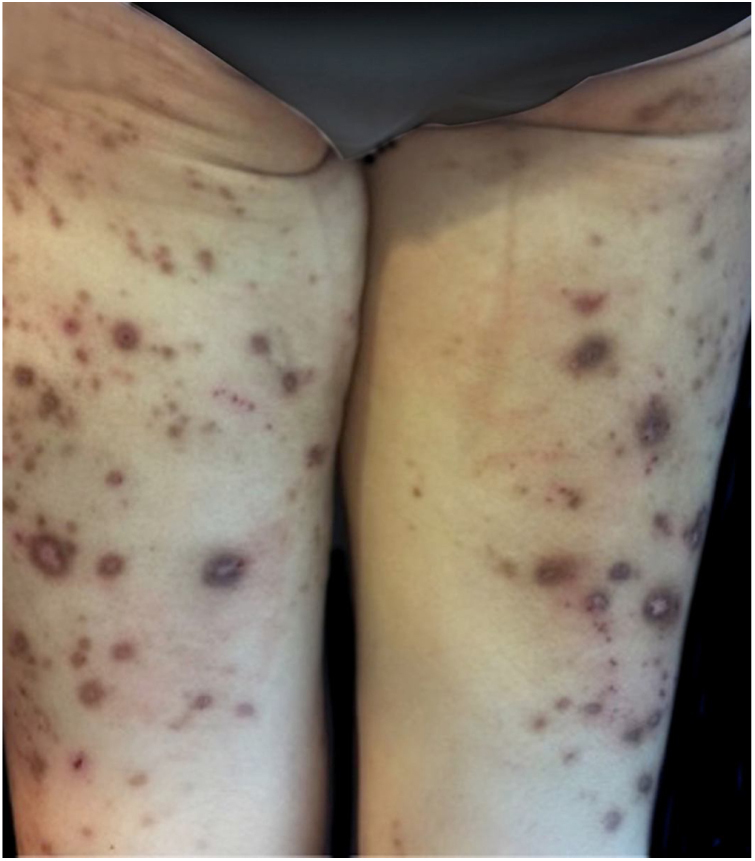
Clinical presentation of a 65-year-old female with generalized umbilicated, hyperpigmented, and excoriated papules and nodules.

**Figure 2:**
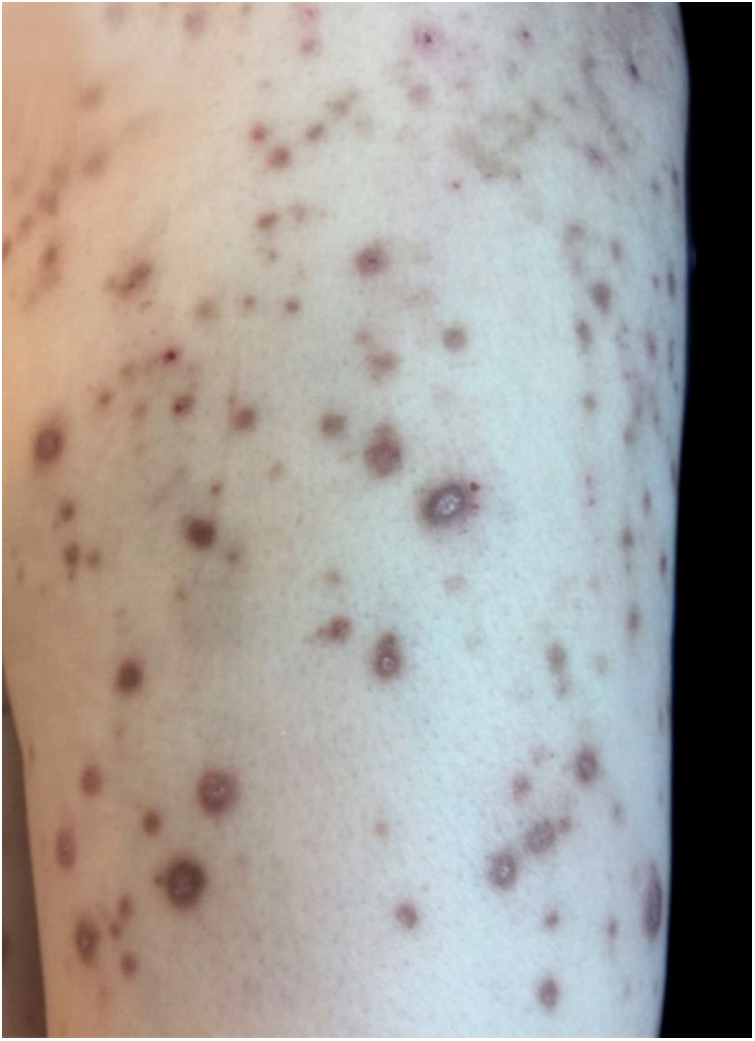
On magnification, a central keratotic plug is visible.

On physical examination, multiple umbilicated, hyperpigmented papules with a central keratotic plug were observed (Figure 1, Figure 2), as well as inguinal and cervical lymphadenopathies measuring up to 2 cm in diameter. Mucous membranes were unaffected.

Laboratory tests revealed mild anemia with mild eosinophilia (hemoglobin 10.9 g/dL, 940 eosinophils/mL), elevated erythrocyte sedimentation rate (93 mm/h), and elevated lactate dehydrogenase (1000 units/L).

## What is your diagnosis?

a) Prurigo nodularis
b) Lichenoid drug eruption
c) Lymphomatoid papulosis
d) Perforating dermatosis

**Figure 3:**
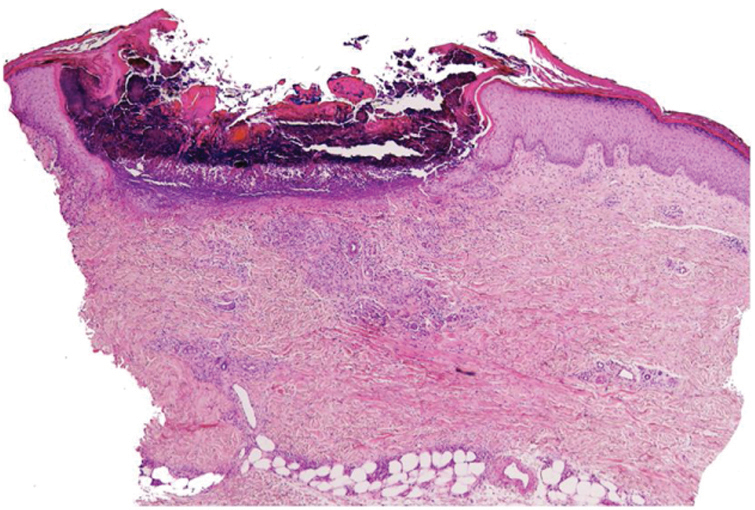
Photomicrograph showing a cup-shaped depression of the epidermis, with an overlying keratin plug containing collagen fibers, keratinous debris, and inflammatory cells (Hematoxylin & eosin, ×100).

**Figure 4:**
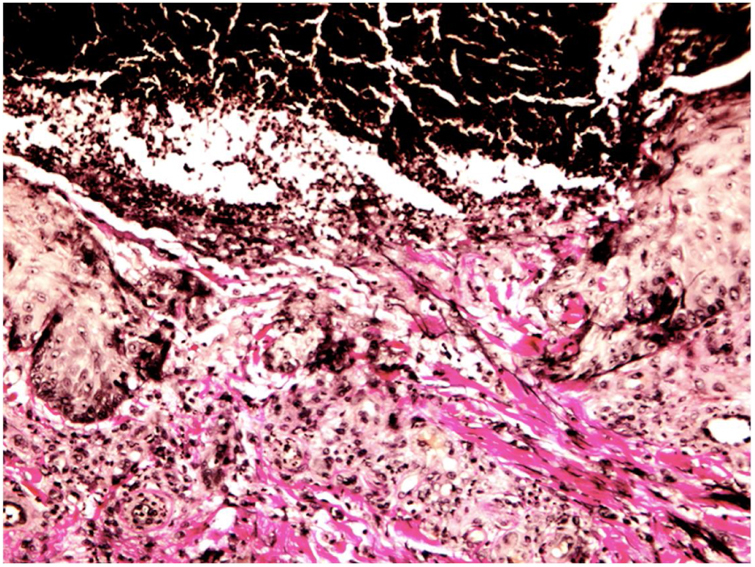
Photomicrograph showing vertically oriented collagen fibers extruding through the epidermis (Van Gieson, ×400).

Skin biopsy showed a cup-shaped depression of the epidermis, with an overlying keratin plug containing collagen fibers, keratinous debris, and inflammatory cells on H&E stained sections. Van Gieson staining demonstrated vertically oriented collagen fibers extruding through the epidermis (Figure 3, Figure 4).

The patient was treated with antihistamines and triamcinolone. A secondary study for a myeloproliferative neoplasm was negative; she was referred to an endocrinologist to improve metabolic management.

## Discussion

Reactive perforating collagenosis (RPC) is a rare disease in the spectrum of perforating dermatoses, showing epidermal perforation and transepidermal elimination of collagen and/or elastic fibers as histologic features.^1^

RPC may be classified into hereditary and acquired forms. The hereditary type appears in early childhood and is genetically determined by autosomal inheritance, whereas the acquired form (ARPC) accompanies systemic diseases, most commonly diabetes mellitus (DM) and chronic kidney disease (CKD).^2^ However, ARPC has also been associated with both myeloproliferative and solid neoplasms.^3^

Clinically, the disease presents with erythematous and/or hyperpigmented papules, plaques, and nodules. Lesions present a central umbilicated or crateriform hyperkeratotic plug, are intensely pruritic, and the Koebner phenomenon is observed. After healing, atrophic, hypo- or hyperpigmented scars are common.^3^, ^4^ These lesions appear in areas of superficial trauma and are most likely due to itching. In diabetic patients, vasculopathy of the dermis has been proposed as a synergistic factor.^5^ The palmoplantar region, intertriginous areas, and mucous membranes are generally unaffected.^3^, ^6^

RPC is a clinical diagnosis that requires histopathological confirmation, and its features depend on the stage of the disease. Initially, degenerate collagen fibers and necrosis are seen extending into the reticulated dermis; epidermal hyperplasia may also be present. In more advanced lesions, the epidermis develops a cup-shaped depression with an overlying basophilic keratin plug consisting of inflammatory cells and keratinous debris. Vertical collagen fibers, which stain red with elastic Van Gieson staining and blue with Masson's trichrome staining, can be observed on the base of the ulcer and extruding through the epidermis.^7^

Treatment goals are improvement of the pruritus and skin lesions and, most importantly, management of associated internal diseases. Primary therapy based on topical corticosteroids, antihistamines, or antibiotics has been recommended. In case of failure, second-line therapy with allopurinol should be considered.^8^, ^9^

This case emphasizes the need to consider a diagnosis of ARPC when faced with chronic pruritic lesions, especially in the context of DM and CKD. However, even in this scenario, when clinical suspicion for an associated neoplasm is high, a basic study for internal malignancies must be performed.

## Financial support

None declared.

## Authors’ contributions

Catalina Hasbún: Approval of the final version of the manuscript; design and planning of the study; drafting and editing of the manuscript; collection, analysis, and interpretation of data; effective participation in research orientation; critical review of the literature; critical review of the manuscript.

Mauricio Sandoval: Approval of the final version of the manuscript; design and planning of the study; intellectual participation in propaedeutic and/or therapeutic conduct of the studied cases; critical review of the manuscript.

Sergio González-Bombardiere: Approval of the final version of the manuscript; collection, analysis, and interpretation of data; intellectual participation in propaedeutic/therapeutic conduct of the studied cases.

## Conflicts of interest

None declared.
